# Optimal Adherence to a Mediterranean Diet May Not Overcome the Deleterious Effects of Low Physical Fitness on Cardiovascular Disease Risk in Adolescents: A Cross-Sectional Pooled Analysis

**DOI:** 10.3390/nu10070815

**Published:** 2018-06-25

**Authors:** César Agostinis-Sobrinho, Rute Santos, Rafaela Rosário, Carla Moreira, Luís Lopes, Jorge Mota, Arvydas Martinkenas, Antonio García-Hermoso, Jorge Enrique Correa-Bautista, Robinson Ramírez-Vélez

**Affiliations:** 1 School of Physical Education, Physiotherapy and Dance, Federal University of the South of Brazil, Porto Alegre 91501-970, Brazil; cesaragostinis@hotmail.com; 2 Research Centre in Physical Activity, Health and Leisure, Faculty of Sport, University of Porto, 4200450 Porto, Portugal; rutemarinasantos@hotmail.com (R.S.); carla_m_moreira@sapo.pt (C.M.); luis.iec.um@hotmail.com (L.L.); jmota@fade.up.pt (J.M.); 3 Faculty of Health Sciences, Klaipeda University, LT-91274 Klaipeda, Lithuania; Arvydas.Martinkenas@ku.lt; 4 Early Start Research Institute, Faculty of Social Sciences, School of Education, University of Wollongong, Wollongong, NSW 2522, Australia; 5 School of Nursing, Research Centre in Child Studies, University of Minho, 4710 Braga, Portugal; rrosario@ese.uminho.pt; 6 Laboratorio de Ciencias de la Actividad Física, el Deporte y la Salud, Facultad de Ciencias Médicas, Universidad de Santiago de Chile, USACH, Santiago 7500618, Chile; antonio.garcia.h@usach.cl; 7 Centro de Estudios Para la Medición de la Actividad Física CEMA, Escuela de Medicina y Ciencias de la Salud, Universidad del Rosario, Bogotá 111221, Colombia; jorge.correa@urosario.edu.co

**Keywords:** cardiometabolic health, diet patterns, aerobic fitness, muscular strength, youth

## Abstract

To examine the combined association of cardiorespiratory fitness (CRF), muscular fitness (MF), and adherence to a Mediterranean diet (MeDiet) on cardiovascular risk in adolescents, a pooled study, including cross-sectional data from two projects [2477 adolescents (1320 girls) aged 12–18 years], was completed. A shuttle run test was used to assess CRF. MF was assessed by the standing-long jump and handgrip tests. Adherence to a MeDiet was assessed by the Kidmed questionnaire. A cardiovascular risk score was computed from the following components: Age and sex, waist circumference, triglycerides, systolic blood pressure, high-density lipoprotein cholesterol (HDL), and glucose. Analysis of covariance showed that participants classified as having optimal (High) adherence to a MeDiet/HighMF/HighCRF, as well those classified as low adherence to a MeDiet/HighMF/HighCRF, had, on average, the lowest cardiovascular risk score (F = 15.6; *p* < 0.001). In addition, the high adherence to a MeDiet/LowMF/LowCRF group had the highest odds of having a high cardiovascular risk (OR = 7.1; 95% CI: 3.4–15.1; *p* < 0.001), followed by the low adherence to a MeDiet/LowMF/LowCRF group (OR = 3.7; 95% CI: 2.2–6.3; *p* < 0.001), high adherence to a MeDiet/HighMF/LowCRF group (OR = 3.1; 95% CI: 1.4–7.0; *p* = 0.006), and low adherence to a MeDiet/LowMF/HighCRF group (OR = 2.5; 95% CI: 1.5–4.4; *p* = 0.002) when compared to those with high adherence to a MeDiet/HighMF/HighCRF, after adjustments for potential confounders. In conclusion, our findings showed that, regardless of the MeDiet status, adolescents with low MF and low CRF cumulatively, presented the highest cardiovascular disease risk. Therefore, these findings suggest that the combination of these two fitness components may be beneficial to adolescents’ cardiometabolic profile, independent of MeDiet behaviour.

## 1. Introduction

Cardiometabolic disorders in children and adolescents are occurring progressively at early ages worldwide [1]. From a public health perspective, this is of great concern due to the tracking of childhood metabolic syndrome into adulthood [2]. The most accepted cardiometabolic risk factors during childhood and adolescence are triglycerides, blood pressure, high-density lipoprotein cholesterol (HDL), total cholesterol (TC), waist circumference, and insulin resistance [3,4].

Adolescence is a crucial period in life for the adoption of lifestyle patterns that are likely to track into their adulthood and, because many chronic diseases that appear in adulthood have their roots in childhood, it is important for youth to establish healthy habits early in life. Although a number of non-modifiable factors influence cardiometabolic health, it can be improved with healthful dietary habits and ideal levels of physical activity during childhood and adolescence [5]. Unfortunately, there is well-documented evidence on poor diet quality [6,7,8], inadequate physical activity levels [9,10], and the increase in the prevalence and severity of metabolic syndrome [11] in early ages.

Physical fitness is recognized as an independent predictor of cardiovascular disease (CVD) and adulthood mortality [12,13]. Physical inactivity and low physical fitness levels, particularly cardiorespiratory fitness (CRF) [14,15,16] and muscular fitness (MF) [17,18], have been associated with cardiovascular risk factors in adolescents, such as insulin resistance, obesity, high blood pressure, and lipid disorders, among others. 

Studies in adolescents have investigated the association of individual physical fitness elements with cardiovascular risk factors [15,16,19]. Oftentimes, both MF and CRF have been mainly treated as an independent variable [19,20] and only a few studies have investigated the combination of both components [21,22].

A growing body of evidence strongly supports the assumption that an adherence to a Mediterranean diet pattern may reduce the risk of cardiometabolic and non-communicable diseases from early ages [23]. The Mediterranean diet (MeDiet) is characterized by being poor in saturated fat and rich in natural antioxidants, based on the consumption of fish, vegetables, fruits, legumes, olive oil, and nuts [23]. Previous studies have shown a positive relationship between an adherence to the MeDiet and cardiovascular health in adolescents [24].

Although the effect of MeDiet, CRF, and MF have already been individually associated to cardiometabolic outcomes, the influence of the combination of these three health determinants remain unknown in adolescents. To our knowledge, there are no data on the combined effect of CRF, MF, and MeDiet on cardiovascular health during adolescence. In this sense, considerable effort and attention are needed to improve the understanding of the combination of these health determinants on cardiovascular health in adolescents. In this context, the present study aimed to examine the combined association of CRF, MF, and adherence to a MeDiet on cardiovascular disease risk in adolescents.

## 2. Methods

### 2.1. Study Design, Sampling Procedures, and Participants

A relatively large-scale study (FUPRECOL study, in Spanish—*Asociación de la fuerza prensil con manifestaciones de riesgo cardiovascular tempranas en niños y adolescentes colombianos*) [25,26] with 1948 adolescents was used and we then complemented this data with another similar project with adolescents of the same age (LabMed Study [25]) to increase the sample size for optimal adherence to a MedDiet (i.e., combined groups with CRF and MF), which is the main focus of this paper. Therefore, the current report is part of two studies: (1) The “Longitudinal Analysis of Biomarkers and Environmental Determinants of Physical activity (LabMed Physical Activity Study)”, a school-based prospective cohort study carried out in four Portuguese cities from the North Region in adolescents from 12–18 years old. Detailed descriptions of the sampling and recruitment approaches and data collection have been described elsewhere in detail [27,28]. The LabMed Physical Activity Study was conducted in accordance with the Helsinki Declaration for Human Studies and was approved by the Portuguese Data Protection Authority (#1112434/2011) and the Portuguese Ministry of Science and Education (0246200001/2011); and (2) The FUPRECOL Study is a cross-sectional study that seeks to establish the general prevalence of CVD risk factors (anthropometric, adiposity, metabolic, and genetic markers) in the study population (children and adolescents aged 9 to 17.9 years living in Bogota, Colombia). The FUPRECOL was approved by the institutional review board for the use of human subject research in addition to the Rosario University Board (Code CEI-ABN026-000262). The protocol was in accordance with the current Colombian laws governing clinical research on human subjects (Resolution 008430/1993 of the Ministry of health). Detailed descriptions of the sampling and recruitment approaches and data collection have been described elsewhere [25,26]. 

For the present pooled analysis study, we include 2477 adolescents: 529 were from the LabMed study (267 girls, 262 boys, mean age 14.3 ± 1.7 years), and 1948 from the FUPRECOL study (1053 girls, 895 boys, mean age 14.2 ± 1.5 years). In the current paper, we include only the adolescents that have completed the same tests and methodological approaches for MF, CRF, diet pattern, pubertal stage, and all cardiometabolic variables.

### 2.2. Adherence to the Mediterranean Diet

The adherence to the MeDiet was assessed using the Kidmed index (Mediterranean Diet Quality Index for children and adolescents) [29]. The index is based on a 16-question, self-administered questionnaire, which sustain the principles of the Mediterranean dietary patterns, as well as those that undermine it. The final results of the index varied between 0 and 12 points. A value of 1 was given to the questions which have a positive connotation in accordance to the MeDiet (+1). Participants were classified into the follow three levels: (1) ≤3, very low diet quality; (2) 4–7, improvement needed to adjust intake to Mediterranean patterns; (3) ≥8, optimal (High) adherence to the Mediterranean diet. To further analyses, we grouped level 2 and 1 and this group was classified as Low Adherence to a MeDiet. 

### 2.3. Cardiorespiratory Fitness

CRF was assessed using the 20-m shuttle run test as previously described elsewhere [30]. Participants were asked to run back and forth between two parallel lines spaced 20 m apart, following the pace of an audio signal that began at a speed of 8.5 km/h and increased by 0.5 km/h at 1-min intervals. The maximum oxygen consumption (VO_2_max, mL/kg/min) was estimated from the number of laps performed using the equation reported by Leger et al. [30]. The participants were also classified into two CRF groups (low and high) in accordance with the proposed cut-off for this population by Ruiz et al. [19]. 

### 2.4. Muscular Fitness

We used a handgrip dynamometer, (T.K.K. 5001, Grip-A, Takei, Japan), adjusted by sex and hand size for each adolescent, to assess the upper body isometric strength (handgrip strength test). The adolescents were instructed to stand with their arms completely extended, squeezing gradually and continuously on the handgrip, up to the maximum of their strength, for at least 2 s, performing the test twice and alternating with both hands. A rest period of 90-s was given between trials. The best score for each hand was recorded in kilograms [31]. The handgrip score (kg) was calculated as the average of the left and right and then expressed per kilogram of body weight [28].

To assess the lower body explosive strength, the standing long jump test was performed in an indoor wood floor gymnasium and the adolescents were instructed to jump from the starting line and to push off vigorously and jump as far forward as possible, landing on both feet, and staying upright. The participants performed the test twice and the best jump was recorded. The standing jump score was determined by the distance between the last heel-mark and the take-off line [31].

To create the muscular fitness score, the results of the handgrip strength and standing long jump tests were transformed into *Z*-scores by sex and age for the whole sample and then the sum of both tests was performed. Participants were divided into two groups: High MF group (second and third tertiles) and Low MF group (first tertile) [28].

### 2.5. Data Management

Eight exclusive groups were created according of the adherence to a MeDiet (Low and High), CRF group (Low and High), and MF (Low and High): (1) Low-MeDiet with LowMF/LowCRF; (2) Low-MeDiet with HighMF/LowCRF; (3) Low-MeDiet with LowMF/HighCRF; (4) Low-MeDiet with HighMF/HighCRF; (5) High-MeDiet with LowMF/LowCRF; (6) High-MeDiet with HighMF/LowCRF; (7) High-MeDiet with LowMF/HighCRF; and (8) High-MeDiet with HighMF/HighCRF.

### 2.6. Cardiometabolic Variables

As previously described elsewhere [27,32], blood samples (triglycerides, HDL-C, glucose) and blood pressure were collected after an overnight fast (>10 h) and blood samples were collected according to standardized procedures. All assays were performed in duplicate according to the manufacturers’ instructions. 

Using the above components, we calculated a continuous score representing a composite cardiovascular disease risk score and was derived by summing the standardized values [*Z*-score = (participant’s value − mean value of the sample)/standard deviation)] by age and sex, waist circumference (WC), systolic blood pressure, triglycerides, HDL-C (inverted), and glucose. Participants above 1 standard deviation (SD) of this score were classified as having a high cardiovascular risk.

### 2.7. Pubertal Stage

Pubertal stage (breast and pubic hair development for girls, genital and pubic hair development for boys; ranging from stage I to V) was self-assessed by the participants according to the classification of the criteria of Tanner and Whitehouse [33].

### 2.8. Statistical Analysis

Descriptive data are presented as means and standard deviations. Chi-squares for categorical variables and independent Two-tailed *t*-Tests for continuous variables were used to assess groups’ differences. Analysis of covariance (ANCOVA) with Bonferroni post-hoc multiple comparison tests were used to assess the differences between the mean values of cardiovascular disease risk scores across the eight combined groups. Binary logistic regression models were constructed to verify the odds ratios (OR) of the combined groups of CRF, MF, and adherence to a MeDiet to predict a high cardiovascular risk. All the analyses were adjusted for, age, sex, pubertal stage, and country. Data were analyzed using SPSS (Statistical Package for the Social Sciences for Windows), version 23.0 SPSS Inc., Chicago, IL, USA. A *p* value < 0.05 denoted statistical significance. 

## 3. Results

### 3.1. Study Participants

Descriptive characteristics of the participants according to CRF, MF, and MeDiet status are presented in Table 1. Participants in the Low MeDiet category had significantly lower glucose and HDL-cholesterol, and higher triglycerides, CRF, and MF (*p* < 0.05 for all) compared with those in the High MeDiet category. Both Low MF group and Low CRF groups showed significantly higher body mass index (BMI), WC, triglycerides, and lower HDL-C compared with their peers (*p* < 0.05 for all).

### 3.2. Relationship between Cardiovascular Risk Scores across Combined Groups of Adherence to a MeDiet, Cardiorespiratory Fitness, and Muscular Fitness 

**Figure 1:**
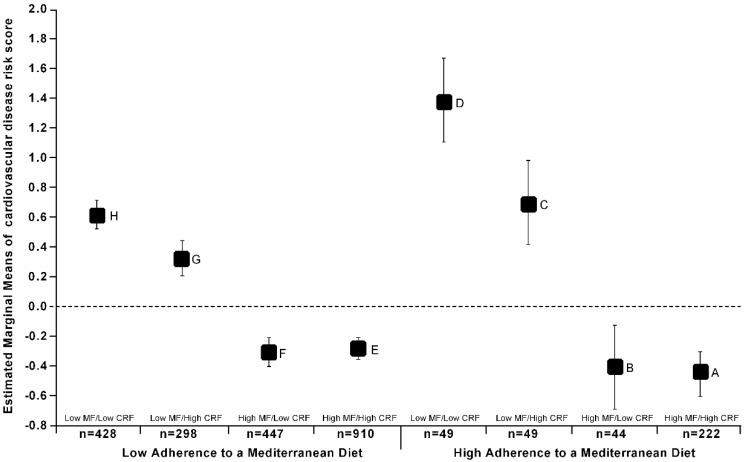
Mean values of cardiovascular risk score through the groups of adherence to a MedDiet (Low adherence vs. High adherence), cardiorespiratory fitness (Low CRF vs. High CRF), and muscular fitness (Low MF vs. High CRF). Bars represent adjusted means and 95% confidence intervals, for age, sex, pubertal stage and country, as confounders. A ≠ (H − G − D − C); B ≠ (D); C ≠ (F − E − A); D ≠ (G − F − E − B − A); E ≠ (H − G − D − C); F ≠ (H − G − D − C); G ≠ (F − E − D − A); H ≠ (F − E − A). *p* < 0.04 for all.

Figure 1 shows the differences in the cardiovascular risk score through the eight exclusive created groups according to the MeDiet, MF, and CRF status. Adolescents classified as having a High-MeDiet and HighMF/HighCRF, as well as the Low-MeDiet and with HighMF/HighCRF group, had, on average, the lowest cardiovascular risk score. In addition, the adolescents classified as having a High-MeDiet and LowMF/LowCRF had, on average, the highest mean values of the cardiovascular risk score (F_(7, 2436)_ = 15.6; *p* < 0.001).

### 3.3. Association between High Cardiovascular Risk by Adherence to a MeDiet, Cardiorespiratory Fitness, and Muscular Fitness Categories

Binary logistic regression analysis showed that participants with a High-MeDiet and LowMF/LowCRF had the highest odds of having a high cardiovascular risk (OR = 7.1; 95% CI: 3.4–15.1; *p* < 0.001), followed by the Low-MeDiet and LowMF/LowCRF group (OR = 3.7; 95% CI: 2.2–6.3; *p* < 0.001), High-MeDiet and HighMF/LowCRF group (OR = 3.1; 95% CI: 1.4–7.0; *p* = 0.006), and the Low-MeDiet and LowMF/HighCRF group (OR = 2.5; 95% CI: 1.5–4.4; *p* = 0.002), when compared to those with High-MeDiet and HighMF/|HighCRF, after adjustments for potential confounders (Table 2). 

## 4. Discussion

In the current study, cardiovascular risk factors, adherence to the MeDiet, MF, and CRF were examined in 2444 adolescents. We found that the combination of low MF and low CRF cumulatively presented the highest cardiovascular disease risk regardless of the MeDiet status. This association persisted after adjustment for age, sex, pubertal stage, and country among participants in the LowMF/HighCRF group and independent of MeDiet status.

Adherence to a healthy dietary pattern, MF, and CRF are critical, yet modifiable, determinants for adolescents’ health-related behaviors. Several studies have reported the beneficial health effects of both MF [28,34,35] and CRF [16,19,36], as well as adherence to a MeDiet [23,24]. However, to our knowledge, there are no data about the combined effect of adherence to a MeDiet, MF, and CRF on cardiovascular risk factors in adolescents.

Indeed, optimal adherence to a Mediterranean dietary pattern is associated with a significantly lower risk of overall mortality, mortality from cardiovascular diseases, incidence of dementia, and mortality from cancer [23]. In adolescents, adherence to a MeDiet has been associated with a healthier metabolic profile [24]. Likewise, our findings showed that the optimal MeDiet group had, on average, an improved metabolic profile when compared with the Low MeDiet group. However, in our combined analysis, the findings suggest that the harmful consequences ascribed to the effect of both low MF and CRF combined could not be counteracted by maintaining of an optimal adherence to a MeDiet. 

Paradoxically, it seems that an optimal adherence of MeDiet, in the presence of both low levels of MF and CRF, may increase cardiometabolic risk. Indeed, a study in Portuguese adolescents showed that fit participants had a lower metabolic risk score, independent of their adherence to a healthy dietary pattern, suggesting that high CRF may overcome the deleterious effects of low adherence to a healthy dietary pattern in adolescents [37]. In addition, previous studies in youth have shown that the MeDiet may have some nutrients, such as sodium and fatty acids, which may contribute, at least in part, to our findings [38].

There is an unequivocal association between low CRF levels and cardiometabolic risk factors in adolescents [36]. Presently, a growing body of evidence has also demonstrated the benefits of ideal levels of MF on metabolic health [34]. Recently, Fraser and colleagues [18], in a longitudinal study of 20 years, demonstrated that childhood MF and CRF were associated with beta cell function and insulin resistance in adulthood. Previously, our group has shown that CRF [15,16,39] and MF [17,20,28] were inversely associated with metabolic risk factors. However, in these studies, the analysis of the combined effect of adherence to a MeDiet and both fitness components on cardiometabolic health was not possible due to the small number of adolescents in the groups. In the current study using pooled analysis, we could show that the Low MedDiet/LowMF/HighCRF group, as well as the optimal MedDiet/LowMF/HighCRF group, might still present a poor cardiometabolic profile. 

Evidence suggests that low MF may be, at least, as important as body composition, [40] and, in some [41,42], but not all studies [43], as important as CRF in determining cardiometabolic risk in adolescents. Indeed, low MF is emerging in the literature as a predictor of mortality and premature death [35,44]. More recently, the potential protective effects of high MF on cardiometabolic health have been found in adults [13], with the suggestion that MF may protect against CVD independently of CRF [12]. Our findings also showed for the first time that adolescents with low MF may display an unhealthy cardiometabolic profile, regardless of CRF and adherence to a MeDiet. In fact, accumulated evidence has suggested that MF might have a stronger influence on the cardiometabolic profile [13,35], although both CRF and MF have showed an independent protection against cardiovascular disease and all-cause mortality in apparently healthy populations [12,13,14]. 

Our findings do support the current literature, which shows both MF and CRF as important markers of cardiometabolic health in youth and adds new information showing the potential health effects of healthy MF and CRF levels to an adherence to a MeDiet. The present study has an important point of view from the public health perspective, since healthy behaviors do not always come together, therefore, active adolescents may have an optimal adherence to a healthy dietary pattern and high levels of physical fitness, but also the opposite [45,46]. Thus, even if an adolescent has an optimal adherence to a MeDiet, it seems that unhealthy levels of physical fitness may increase cardiometabolic risk. These findings highlight the relevance of physical fitness and support the current physical activity recommendations for youths, which include aerobic exercise and muscle strengthening activities [5].

Limitations of our study include its cross-sectional design, which does not allow us to draw any conclusions on the causal direction of the associations. Additionally, the Kidmed questionnaire has inherent limitations of precision due to its reliance on self-reported data, and the choice of research method precluded independent verification [47].

The strengths of our study include the consideration of a fairly large sample size and standardised methods used to collect the data. In addition, the novelty of the analysis of the combined associations of three important health factors on adolescents’ cardiometabolic health.

## 5. Conclusions

On the basis of our results and by accounting for all the limitations inherent in our study, we show that the combination of low MF and low CRF cumulatively presented the highest cardiovascular disease risk regardless of the MedDiet status. Therefore, these findings suggest that the combination of MF and CRF components may be beneficial to adolescents’ cardiometabolic profile, independent of MeDiet behaviour.

## Figures and Tables

**Table 1:** Characteristics of participants (mean (standard deviation (SD)) or frequency (%)).

Characteristics	Low MeDiet (*n =* 2115)	High MeDiet (*n =* 362)	Low MF (*n =* 828)	High MF (*n =* 1649)	Low CRF (*n =* 982)	High CRF (*n =* 1495)
Age (year)	14.2 (1.5)	14.1 (1.6)	14.2 (1.5)	14.2 (1.5)	14.6 (1.5)	14.1 (1.5)
BMI (kg m^2^)	20.3 (3.0)	21.0 (3.7)	21.7 (3.7) ^b^	19.9 (3.1)	21.2 (3.5) ^c^	20.0 (2.7)
Waist circumference (cm)	66.8 (7.6) ^a^	70.8 (10.1)	69.8 (9.6) ^b^	66.2 (7.1)	68.7 (9.2) ^c^	66.5 (7.5)
Pubertal status A: ≤III/IV/V (%)	48.0/44.7/8.3	44.7/48.8/10.5	51.0/41.2/7.8	46.7/44.1/9.2	48.1/42.1/9.8	48.2/43.8/8.1
Pubertal status B: ≤III/IV/V (%)	48.6/43.4/8.0	40.6/43.1/16.3	51.3/41.7.7	46.4/43.0/10.5	49.2/42.0/8.8	47.4/43.0/9.7
HDL-Cholesterol (mg/dL)	47.1 (12.1) ^a^	52.0 (12.2)	45.6 (11.2) ^b^	49.0 (12.6)	46.4 (11.9) ^c^	48,8 (12.5)
Triglycerides (mg/dL)	87.9 (42.1) ^a^	76.6 (37.7)	93.7 (47.6) ^b^	82.5 (37.9)	88.7 (42.8) ^c^	84.6 (40.9)
Glucose (mg/dL)	83.0 (15.1) ^a^	86.4 (12.3)	82.7 (15.0)	83.4 (14.9)	83.0 (15.6)	83.3 (14.5)
Systolic Blood Pressure (mm Hg)	113.1 (13.1)	116.5 (13.8)	114.1 (13.4)	113.3 (13.1)	113.5 (13.4)	113.7 (13.7)
CRF—VO_2_max (mL/kg/min)	39.3 (5.7) ^a^	42.1 (6.4)	37.3 (5.1) ^b^	40.9 (5.9)	-	-
Muscular Fitness Score	−0.8 (1.5) ^a^	0.4 (1.7)	-	-	−0.6 (1.4) ^c^	0.4 (1.5)

Data are means (±standard deviation) or frequencies (%) ^a^ Significantly different from High MedDiet (*p* < 0.05), ^b^ Significantly different from High MF (*p* < 0.05), ^c^ Significantly different from High CRF (*p* < 0.05)—Chi-square for categorical variables and independent Two-tailed *t*-Tests for continuous variable. BMI: Body mass index; MeDiet: Adherence to the Mediterranean diet; MF: muscular fitness; CRF: Cardiorespiratory fitness; Pubertal stage-A—breast development in girls; genital development in boys. Pubertal stage-B—pubic hair development.

**Table 2:** Odds ratio of high cardiovascular risk by adherence to a Mediterranean Diet, cardiorespiratory fitness, and muscular fitness categories.

Parameter	High Cardiovascular Risk			
	OR Unadjusted (95% CI)	*p*-Value	OR Adjusted (95% CI) *	*p*-Value
High-MeDiet HighMF/HighCRF	1.0	-	1.0	-
High-MeDiet HighMF/LowCRF	2.0 (0.8–4.9)	0.109	1.8 (0.7–4.0)	0.165
High-MeDiet LowMF/HighCRF	2.5 (1.1–5.6)	0.023	3.1 (1.4–7.0)	0.006
High-MeDiet LowMF/LowCRF	6.5 (3.1–13.3)	<0.001	7.1 (3.4–15.1)	<0.001
Low-MeDiet HighMF/HighCRF	0.9 (0.6–1.5)	0.897	1.3 (0.7–2.1)	0.346
Low-MeDiet HighMF/LowCRF	1.1 (0.6–1.9)	0.611	1.6 (0.8–2.7)	0.112
Low-MeDiet LowMF/HighCRF	1.5 (0.9–2.6)	0.113	2.5 (1.5–4.4)	0.002
Low-MeDiet LowMF/LowCRF	2.5 (1.5–4.1)	<0.001	3.7 (2.2–6.3)	<0.001

OR, odds ratios; CI, confidence intervals; 1, reference category. * Adjusted for age, sex, pubertal stage, country. MF, Muscular Fitness. CRF, Cardiorespiratory Fitness.

## Abbreviations

The following abbreviations are used in this manuscript:

BMI	Body mass index
CRF	Cardiorespiratory fitness
CVD	Cardiovascular disease
HDL	High-density lipoprotein cholesterol
MedDiet	Adherence to Mediterranean Diet
MF	Muscular Fitness
TC	Total cholesterol
VO_2_max	Maximum oxygen consumption
WC	Waist circumference
